# Congenital toxoplasmosis in infants from chronically infected mothers: report of two cases

**DOI:** 10.1590/1984-0462/2025/43/2024120

**Published:** 2025-01-17

**Authors:** Roberta Rassi Almeida, Geovana Batista de Campos, Ana Maria Castro

**Affiliations:** a Universidade Federal de Goiás, Institute of Tropical Pathology and Public Health, Parasite-Host Relationship Studies Laboratory, Goiânia, GO, Brazil.

**Keywords:** Child, Congenital toxoplasmosis, Chronic infection, Neglected diseases, Case report, Criança, Toxoplasmose congênita, Infecção persistente, Doenças negligenciadas, Relato de caso

## Abstract

**Objective:**

To describe two severe cases of congenital toxoplasmosis in infants born to chronically infected mothers who did not receive education or information on the prevention of gestational toxoplasmosis during prenatal care.

**Case description:**

The mothers had a previous serological diagnosis of toxoplasmosis conducted during prenatal care, with non-reactive (<10 IU/mL) IgM and reactive IgG (>10 IU/mL), and were considered “immune” to the infection. Both infants were born with sequelae of the congenital infection, including neurological and ocular alterations.

**Comments:**

Managing gestational toxoplasmosis in susceptible pregnant women is a considerable challenge in several countries, especially in South America. It is necessary to diagnose and monitor chronic gestational toxoplasmosis, as it may result from reactivation or reinfection. Both forms can cause sequelae and irreparable damage to newborns. In addition, it is essential to guide all pregnant women on how to avoid contact with *Toxoplasma gondii*, regardless of their serological status.

## RESUMO

**Objetivo:**

Descrever dois casos graves de toxoplasmose congênita em crianças nascidas de mães cronicamente infectadas que não receberam orientação sobre a prevenção da toxoplasmose gestacional durante o pré-natal.

**Descrição do caso:**

As mães tinham sorologia prévia para toxoplasmose, realizada durante o pré-natal, com IgM não reagente (<10 UI/mL) e IgG reagente (>10 UI/mL) e foram consideradas “imunes” à infecção. Ambos os neonatos nasceram com sequelas da infecção congênita, incluindo alterações neurológicas e oculares.

**Comentários:**

O combate à toxoplasmose gestacional em gestantes suscetíveis é um desafio em vários países, especialmente na América do Sul. É necessário diagnosticar e monitorar a toxoplasmose gestacional crônica, pois ela pode ser decorrente de reativação ou reinfecção. Ambas as formas podem causar sequelas e danos irreparáveis aos recém-nascidos. Além disso, é essencial orientar todas as gestantes sobre como evitar o contato com *Toxoplasma gondii*, independentemente da sorologia.

## INTRODUCTION

Toxoplasmosis is a cosmopolitan zoonosis that affects humans and is often neglected. Transmission can occur by ingesting contaminated water and food. The vertical form happens because the tachyzoites can migrate transplacentally.^1^ According to Rostami et al., the seroprevalence of antibodies against *Toxoplasma gondii* tends to increase with age and varies according to geographical location, health education, hygiene, eating habits, and climatic conditions. The congenital form of toxoplasmosis requires attention as it can have clinical repercussions ranging from asymptomatic to mild or severe symptoms and even death.^2-4^ The ocular lesions caused by congenital toxoplasmosis are usually more severe and more frequent in children from South America. Particularly, Brazil has a high morbidity of congenital toxoplasmosis.^5^

A paradigm about toxoplasmosis has been discussed in recent years: pregnant women with chronic *T. gondii* infection infect their infants. Although uncommon, vertical transmission of toxoplasmosis from chronically infected pregnant women to their fetuses has been documented in humans. Studies suggest that transmission may occur in 0–6% of pregnancies involving immune-competent, chronically infected mothers.^6^

Cases of congenital toxoplasmosis resulting from maternal reinfection or reactivation of a chronic infection have been reported. This highlights the importance of these events in studies on congenital toxoplasmosis.^7^ Assessing the risk of vertical transmission is crucial for providing clinical counseling and guiding treatment decisions for pregnant women and infants. Factors such as gestational age at maternal seroconversion and specific antibody profiles are used to estimate the risk of transmission.^8^ Although rare, it is crucial to continuously monitor and assess the risk of vertical transmission from chronically infected mothers throughout pregnancy.

This study aimed to describe two severe cases of congenital toxoplasmosis in infants born to chronically infected mothers who did not receive guidance on the prevention of gestational toxoplasmosis during prenatal care. The participants signed a free and informed consent form (Plataforma Brasil, #5.208.385).

## CASE REPORT

### Case description – Report 1

The infant was brought to the health service at 11 months old. Prior data include: female newborn from a mother in her third pregnancy, delivered by cesarean section, with a gestational age of 37 weeks, birth weight of 2270 g, height of 43 cm, and head circumference of 30 cm. The neonatal anthropometric data percentiles were below the expected levels: weight <P3, height <P3, and head circumference <P3.

The vaccination booklet did not describe any changes in the physical examination at birth. The results of the fetal ultrasound during pregnancy were normal according to the mother, but they were not shown at the appointments. Cerebrospinal fluid was not collected because the infant arrived at the health service at 11 months old without treatment and with a ventriculoperitoneal shunt (performed at 6 months old).

The mother was 30 years old, had no previous immunodeficiency, and tested non-reactive human immunodeficiency virus (HIV) serology during prenatal care. The mother had serological diagnosis of toxoplasmosis in an earlier pregnancy in May 2017, showing non-reactive IgM and reactive IgG. A serological test during prenatal care in June 2019 detected non-reactive IgM and reactive IgG (>200 IU/mL). The pregnant woman was in her seventh week of pregnancy when this serological test was conducted.

The same laboratory conducted both serological tests using the enzyme-linked immunosorbent assay method. No serological test for toxoplasmosis was performed at the maternity hospital where the infant was delivered. The infant’s serological test was conducted at 7 months of age, showing IgM reactivity at 17.27 IU/mL and IgG reactivity at 43.10 IU/mL. The infant had not been treated until then. At 11 months, upon arrival at the health service, treatment with sulfadiazine, pyrimethamine, and folinic acid was initiated. The mother stated that she could only follow up at that time due to the COVID-19 pandemic. The infant had a poor prognosis, with neurological and ophthalmological sequelae, low weight, and short stature. A multi-professional team monitored the infant, although she started late.

**Figure 1:**
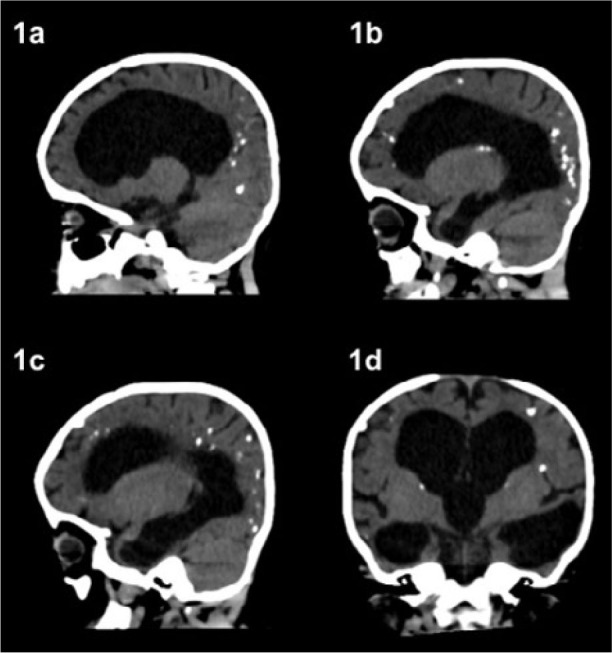
Case report number 1. Brain tomography performed when the infant was 1 year and 3 months old. Supratentorial asymmetrical hydrocephalus, with reduced amplitude of the cerebral aqueduct and IV ventricle, multiple calcifications scattered throughout the brain, and encephalomalacia.

When the infant was approximately two months old, the mother noticed that the infant had strabismus and could not follow objects. An ophthalmologist diagnosed chorioretinitis in both eyes due to probable congenital toxoplasmosis. The neurologist monitored the infant, and a computed tomography scan revealed asymmetrical hydrocephalus in the supratentorial region. There was a reduced amplitude of the cerebral aqueduct and IV ventricle, multiple calcifications scattered throughout the brain, and encephalomalacia (Figure 1). At six months old, the infant experienced a seizure and had to be submitted to a ventriculoperitoneal shunt surgery. Despite all these findings, no treatment was initiated.

Neurological and ophthalmological sequelae are described below, including the last assessment:

- 1 year and 5 months old. Fundoscopy findings in the right eye: calm, clear cornea, formed anterior chamber, and nystagmus with non-viable pupillary secretion. Fundoscopy findings in the left eye: calm, clear cornea, formed anterior chamber, nystagmus, pale optic nerve, and extensive inactive macular lesion.
- 4 years and 6 months old. Head tomography findings: intraparenchymal calcifications without a halo of edema or focal mass effect, varying in dimensions and dispersed in both cerebral hemispheres, along with an increased volume of the lateral ventricles; fourth ventricle with normal morphology and dimensions; cerebellum and brainstem also with normal morphology and attenuation values; and residual calcifications present in both cerebral hemispheres, indicating supratentorial hydrocephalus.

### Case description – Report 2

The male infant was delivered by cesarean section with a pelvic presentation. He was born at a gestational age of 38 weeks and 3 days, weighing 2400 g, measuring 45 cm in length, and head circumference of 31 cm. The percentiles of neonatal anthropometric data of the head circumference were <P3. No abnormalities were described during the physical examination at birth. The fetal ultrasound results in the first and third trimesters showed no changes.

The infant was born to a 37-year-old mother in her fifth pregnancy, with no previous immunodeficiency and non-reactive HIV serology during prenatal care and in the maternity ward. This mother did not have any serological diagnosis of toxoplasmosis from previous pregnancies for comparison, as her youngest child was 15 years old, and prenatal care had been carried out in another state. The mother did not bring the prenatal care test results to the maternity ward, so they had to be requested again.

Therefore, during the postpartum period, the mother had a serological diagnosis of toxoplasmosis, which showed reactive IgM and reactive IgG using the Chemiluminescence Microparticle Immunoassay (CMIA) methodology. The tests conducted during prenatal care were requested for comparison. Prenatal serological test for toxoplasmosis was conducted in May 2021 using the immunoenzymatic methodology: IgM was non-reactive (<10 IU/mL), whereas IgG was reactive (20 IU/mL). The woman was in her ninth week of pregnancy at the time of this serological test. Maternal serological test was conducted postpartum, and the results showed IgM-reactive (12.83 IU/mL) and IgG-reactive (>200 IU/mL).

**Figure 2:**
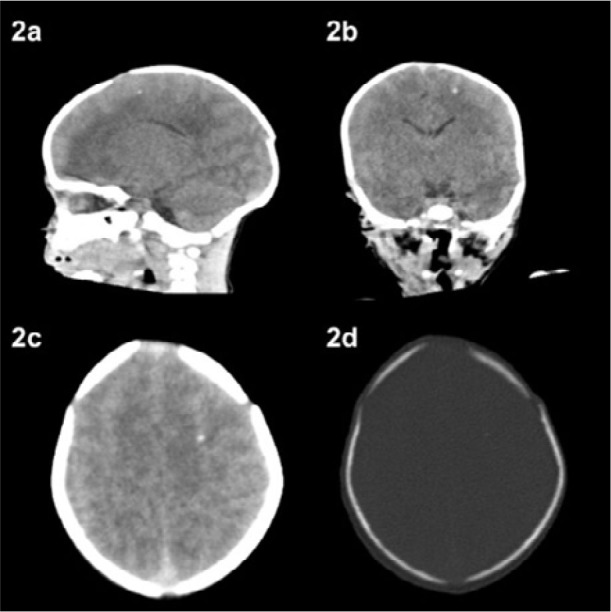
Case report number 2. Brain tomography performed in the maternity ward. Calcification in the subcortical region in the left cerebral hemisphere.

In the maternity ward, the investigation of the newborn was initiated due to reactive IgM and IgG maternal serology, resulting in a diagnosis of congenital toxoplasmosis with both reactive IgM and IgG in the newborn. The investigation commenced with a computed tomography scan revealing some foci of calcification in a primarily subcortical region in the left cerebral hemisphere (Figure 2).

A fundoscopy showed a macular lesion in the right eye and a pigmented lesion in the temporal arch of both eyes. The cerebrospinal fluid result was within normal limits. Sulfadiazine, pyrimethamine, folinic acid, and prednisone were administered on the second day of life. The infant received prednisolone due to a change in fundoscopy, guided by the Ophthalmology team.

The infant was treated with sulfadiazine, pyrimethamine, and folinic acid for one year. The patient had healed eye lesions and developmental delays compared to his age group. At the time of writing, he was under investigation for autism spectrum disorder.

Neurological and ophthalmological sequelae are described below, including the last assessment:

- 1 year and 4 months old. The ophthalmologist decided to keep the infant under observation. He found a foveal sickness in the right eye, which impaired the visual prognosis of this eye, and a sickness in the upper arcade with a free posterior pole in the left eye. A better visual prognosis was expected for the left eye. At the time of writing, the infant had been monitored for visual stimulation.
- 2 years old. Computed tomography findings: within normal limits; there were no calcifications in the brain.

## DISCUSSION

Pregnant women should receive guidance during prenatal care on all forms of prevention and care to avoid congenital toxoplasmosis. The *Manual de Gestação de Alto Risco* (“High-Risk Pregnancy Manual”) recommends providing specific guidance to all pregnant women to prevent *T. gondii* infection, including those previously diagnosed with chronic infection.^9^ This study presented two cases in which both mothers with chronic toxoplasmosis, non-reactive IgM and reactive IgG during prenatal care, had their infants severely infected with *T. gondii.*

None of the mothers received any information about preventing the disease during prenatal care due to their previous serological status. They were all unaware of this zoonosis. In both cases, the infants did not exhibit specific symptoms of the infection at birth, except for being small for their gestational age. The low birth weight could have been attributed to the congenital infection.

In Brazil, toxoplasmosis seroprevalence in the adult population ranges from 40 to 80%. Several factors are linked to vertical transmission, leading to congenital toxoplasmosis. Factors such as parasitemia, the genotype of *T. gondii*, the gestational age at which the first infection occurred, reaggravation and/or reinfection, treatment during pregnancy, and whether the pregnant woman has any immunodeficiency will define the prognosis.^10,11^

The most significant risk of fetal infection occurs during the primary infection in pregnancy, and the severity of the infection is inversely proportional to the gestational age at the time of maternal infection.^12^ The literature also describes cases of reaggravation and/or reinfection in chronic infections, mainly occurring in pregnant women with primary or secondary immunodeficiency, leading to fetal infection.^13^ Reinfections occur when there is exposure to numerous parasites, a more virulent strain, and/or a parasite of a different genotype.^14^ According to Andrade et al., the reinfection’s impact on the occurrence of congenital toxoplasmosis is unknown.^13,15^

Reactivation is characterized by elevated IgG titers without the presence of IgM, often associated with acute inflammatory ophthalmic lesions, primarily retinochoroiditis.^15^ Although vertical transmission is not common in mothers chronically infected with *T. gondii*, there are several reports in the literature from different geographical locations. According to Elbez-Rubinstein et al., there is a very low probability of reinfection with different genotypes on the European continent. However, in Brazil, congenital toxoplasmosis due to reinfection during pregnancy might not be so exceptional.^16^

In 2010, a case was described in Brazil involving a chronically infected pregnant woman with reactivation of the ocular form and non-reactive HIV serology. She had a fetus with congenital toxoplasmosis.^13^ In 2015, Avelar et al. reported a case of fetal death due to toxoplasmosis in a pregnant woman with chronic infection. She had no history of immunosuppression and non-reactive HIV serology.^17^ Silva et al. described a case of a mother with negative HIV serology who had two children two years apart, and both had symptomatic congenital toxoplasmosis.^18^ All these cases emphasize the importance of prenatal care for chronically infected mothers. Since it is an asymptomatic or oligosymptomatic infection at birth, most cases of congenital toxoplasmosis without prior serological screening will go undetected.

In Brazil, there is currently no centralized program to combat gestational and congenital toxoplasmosis due to the enormous diversity between states. Few states, such as Minas Gerais, offer specific and well-defined propaedeutic and therapeutic plans and protocols for both gestational and congenital toxoplasmosis. Unfortunately, there are still many states that lack such programs.

The reported cases reinforce the importance of training and updating healthcare professionals in the management of toxoplasmosis during prenatal care. The absence of adequate guidance on prevention for chronically infected mothers highlights a gap in the education and training of the professionals involved. The lack of information, even when serologies indicate chronic infection, exposes pregnant women to a high risk of vertical transmission. Healthcare professionals must be able to identify, monitor, and advise pregnant women on infection prevention and management strategies to reduce the sequelae associated with congenital toxoplasmosis.

These two reports aimed to emphasize that congenital toxoplasmosis causes significant sequelae and issues in newborns. Measures to prevent the disease must be addressed and strongly promoted throughout prenatal care. The combat against gestational toxoplasmosis in susceptible pregnant women in South America, particularly in Brazil, is a challenge. It is necessary to diagnose and monitor chronic gestational toxoplasmosis, whether it is due to reactivation or reinfection. Both forms can cause sequelae and irreparable damage to newborns.

## Data Availability

The database that originated the article is available with the corresponding author. CAAE: 53111321.2.0000.5078

## References

[1] Tenter AM, Heckeroth AR, Weiss LM (2000). Toxoplasma gondii: from animals to humans. Int J Parasitol.

[2] Peyron F, L’ollivier C, Mandelbrot L, Wallon M, Piarroux R, Kieffer F (2019). Maternal and congenital toxoplasmosis: diagnosis and treatment recommendations of a French multidisciplinary working group. Pathogens.

[3] Rostami A, Riahi SM, Contopoulos-Ioannidis DG, Gamble HR, Fakhri Y, Shiadeh MN (2019). Acute Toxoplasma infection in pregnant women worldwide: a systematic review and meta-analysis. PLoS Negl Trop Dis.

[4] McAuley JB (2014). Congenital toxoplasmosis. J Pediatric Infect Dis Soc.

[5] Lago EG, Endres MM, Scheeren MF, Fiori HH (2021). Ocular outcome of Brazilian patients with congenital toxoplasmosis. Pediatr Infect Dis J.

[6] Holliman RE, Raymond R, Renton N, Johnson JD (1994). The diagnosis of toxoplasmosis using IgG avidity. Epidemiol Infect.

[7] Kodjikian L, Wallon M, Fleury J, Denis P, Binquet C, Peyron F (2005). Ocular manifestations in congenital toxoplasmosis. Graefes Arch Clin Exp Ophthalmol.

[8] Vimercati A, Greco P, D’Apolito A, Angelici MC, Possenti A, Carbonara S (2000). Risk assessment of vertical transmission of Toxoplasma infections. Acta Biomed Ateneo Parmense.

[9] Brasil. (2022). Departamento de Ações Programáticas. Manual de gestação de alto risco..

[10] Dunn D, Wallon M, Peyron F, Petersen E, Peckham C, Gilbert R (1999). Mother-to-child transmission of toxoplasmosis: risk estimates for clinical counseling. Lancet.

[11] Remington JS (2011). Infectious diseases of the fetus and newborn infant..

[12] Teimouri A, Mohtasebi S, Kazemirad E, Keshavarz H (2020). Role of Toxoplasma gondii IgG avidity testing in discriminating between acute and chronic toxoplasmosis in pregnancy. J Clin Microbiol.

[13] Andrade GM, Vasconcelos-Santos DV, Carellos EV, Romanelli RM, Vitor RW, Carneiro AC (2010). Toxoplasmose congênita em filho de mãe cronicamente infectada com reativação de retinocoroidite na gestação. J Pediatr (Rio J).

[14] Lebas F, Ducrocq S, Mucignat V, Paris L, Mégier P, Baudon JJ (2004). Toxoplasmose congénitale: un nouveau cas d’infection pendant la grossesse chez une femme antérieurement immunisée et immunocompétente. Arch Pediatr.

[15] Brasil. (2018). Protocolo de notificação e investigação: toxoplasmose gestacional e congênita..

[16] Elbez-Rubinstein A, Ajzenberg D, Dardé ML, Cohen R, Dumètre A, Yera H (2009). Congenital toxoplasmosis and reinfection during pregnancy: case report, strain characterization, experimental model of reinfection, and review. J Infect Dis.

[17] Avelar JB, Rezende HH, Storchilo HR, Candido RR, Amaral WN, Avelino MM (2015). Reativação da toxoplasmose durante o oitavo mês de gestação.. Revista Norte Mineira de Enfermagem..

[18] Silva MS, Yamamoto AY, Carvalheiro CG, Grigg ME, Medeiros AL, Mussi-Pinhata MM (2021). Congenital ocular toxoplasmosis in consecutive siblings. Arq Bras Oftalmol.

